# Uncertainty promotes information-seeking actions, but what information?

**DOI:** 10.1186/s41235-020-00245-2

**Published:** 2020-09-07

**Authors:** Ashlynn M. Keller, Holly A. Taylor, Tad T. Brunyé

**Affiliations:** 1 Department of Psychology, Tufts University, 490 Boston Ave., Medford, MA 02155 USA; 2 Tufts University, Center for Applied Brain and Cognitive Sciences, 200 Boston Ave., Suite 1800, Medford, MA 02155 USA; 3 US Army CCDC Soldier Center, 15 General Greene Ave., Natick, MA 01760 USA

**Keywords:** Uncertainty, Spatial learning, Spatial reorientation, Information gathering

## Abstract

Navigating an unfamiliar city almost certainly brings out uncertainty about getting from place to place. This uncertainty, in turn, triggers information gathering. While navigational uncertainty is common, little is known about what type of information people seek when they are uncertain. The primary choices for information types with environments include landmarks (distal or local), landmark configurations (relation between two or more landmarks), and a distinct geometry, at least for some environments. Uncertainty could lead individuals to more likely seek one of these information types. Extant research informs both predictions about and empirical work exploring this question. This review covers relevant cognitive literature and then suggests empirical approaches to better understand information-seeking actions triggered by uncertainty. Notably, we propose that examining continuous navigation data can provide important insights into information seeking. Benefits of continuous data will be elaborated through one paradigm, spatial reorientation, which intentionally induces uncertainty through disorientation and cue conflict. While this and other methods have been used previously, data have primarily reflected only the final choice. Continuous behavior during a task can better reveal the cognition-action loop contributing to spatial learning and decision making.

## Significance

When people go to a new city, they can feel discomfort emanating from uncertainty about how to find their way around. As technology has developed, a response to this uncertainty includes heavy reliance on navigational aids. However, using navigational aids is not without problems. Specifically, research suggests that navigational aids impair environmental learning. Further, this technology is not infallible. The combination of these two issues can limit people’s ability to navigate their environment. Uncertainty also likely promotes environmental learning, in part through information gathering about the environment. A better understanding about what information people seek when they are spatially uncertain can inform changes in both environment-learning strategies and navigational aid design. Notably, environment-learning strategies and navigational aids could focus on the information people seek and then use when uncertain, potentially reducing one’s information-processing load. This paper proposes that examining continuous behavioral data (e.g., position and heading) during navigation can provide insights into what information people consider when uncertain. We summarize existing uncertainty and navigation research and then offer a first-step method to assess information gathering during navigational uncertainty in a simple environment. This information could lead to navigational aid improvements that promote rather than inhibit environmental learning.

## Introduction

Imagine moving to a new city. You have just opened an account at the local bank and now want to purchase some groceries. It is unlikely that you know the closest grocery store’s location, assuming it is not directly visible. Therefore, as you walk out the bank door, you wonder in which direction you should go. Do you go straight? Left? Right? What information do you seek to decide? Uncertainty, a cognitive state, is common in unfamiliar environments and almost certainly promotes information seeking, an action. You might look left and right, pull out a map (now often on a smart device), or ask a passerby for directions. Notably, spatial information seeking involves a series of actions, e.g., heading in a particular direction, looking around, pulling up maps, etc.

**Fig. 1:**
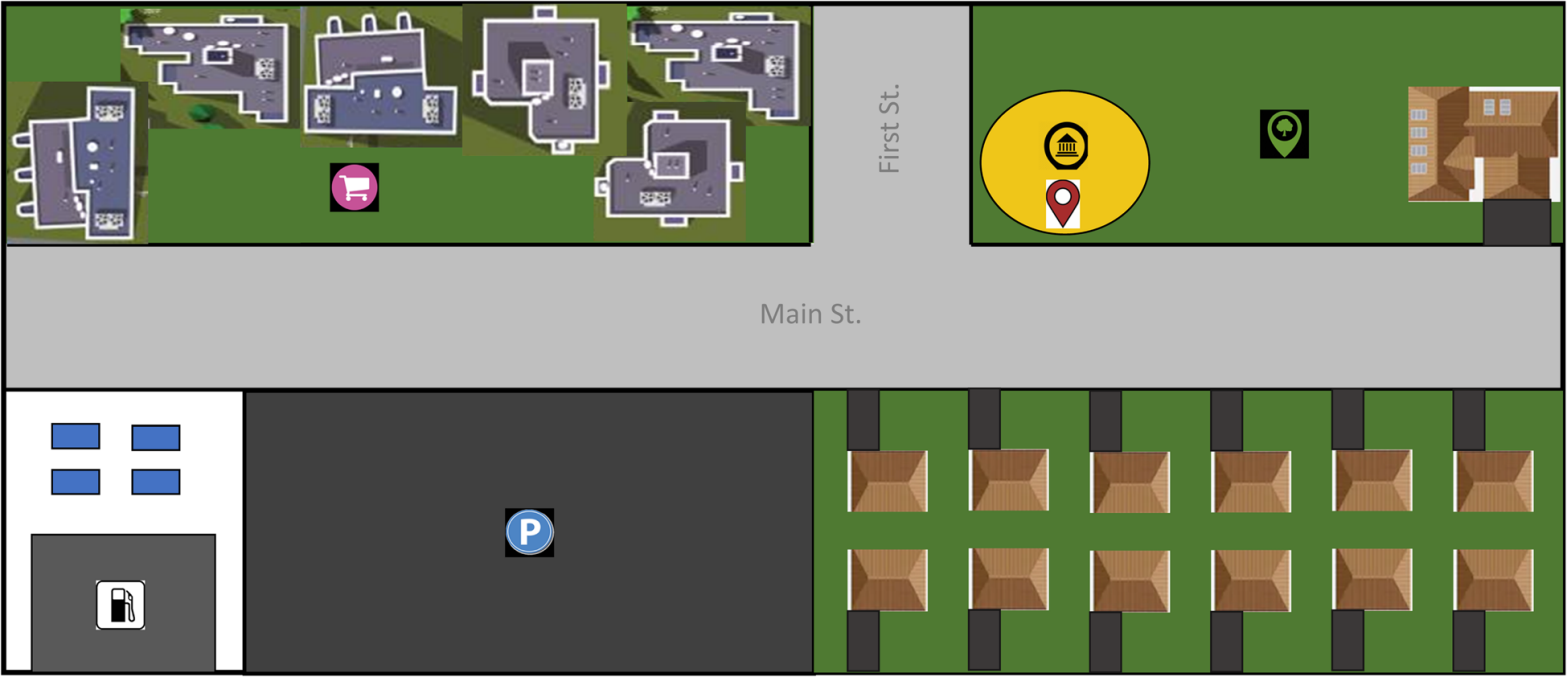
Example community map. Imagine standing at the bank (*yellow oval*) and needing to find a grocery store. *Landmark* information differs in the visual character of different buildings or other spaces. Here landmark information could be the parking lot, a house, or the shopping center. *Landmark configuration* can be seen in the relative density or layout of landmarks. A useful landmark configuration here could be a housing development next to a parking lot which is across the street from a business center. A grocery store is less likely to be in a housing development than a shopping center, and a parking lot is indicative of a lot of traffic (which could be near a shopping center). *Geometric information* relates to the shape or layout of the entire environment and here can be examined globally as the grid-like layout of the streets on this map or locally with the rectangular layout of the business center. Although the figure gives only a snapshot of the community, its rectangular form reflects the overall environment geometry

Information-seeking actions can be directed to different types of environmental information. Return to your quest of finding the nearest grocery store from the bank. When looking around, you might see only houses to your left, but businesses and more people to your right. Using this information (Dalton, Hölscher, & Montello, 2019), you turn right. At this point, you may also pull up a map (Fig. 1), and after noting what looks like a rectangular business center (geometric information) and a building with a grocery store icon (landmark information), you are more confident in your navigation decision and mentally note the business district’s location. As this scenario illustrates, different types of information can be used to make decisions within environments. Environmental learning involves encoding this information. While navigating, you become aware of specific *landmarks* (e.g., bank, park, parking lot). You may note, on a map or while navigating, how those landmarks form a path or that in some areas they are closer together (*configural information*, including *routes*), likely meaning they are closer to the city center. From a map (Fig. 1) you might register that the business district forms an elongated rectangle oriented east-west and that various residential neighborhoods abut the business district (*geometric information* gathered from a *survey* perspective). While spatial cognition research has variably defined the different spatial information types, here we will primarily focus on *landmark*, *configural*, and *geometric* information when discussing the information people seek when uncertain.

These information types often overlap. Individual landmarks make up configurations, and these configurations may contribute to the overall geometry of the space. Little is known about how frequently we seek one or a combination of information types over others when uncertain and how this impacts our navigational actions and the development of spatial memory. This review explores uncertainty’s role in spatial learning, covering extant behavioral and neural literature to inform predictions about information seeking. We then suggest that continuous behavioral measures can provide insights into information seeking during navigation. While continuous *neural* measures would likely also be informative, and we have used them to inform our ideas, this paper focuses specifically on continuous behavioral measures. We will discuss the benefits of measuring behavioral continuously and then detail how these continuous behavioral measures could be used with a popular spatial navigation paradigm.

Information seeking when uncertain likely aims to weight one choice as more probable. A navigator may seek to accumulate enough environmental information to reach a confidence threshold associated with a decision, such as whether to continue straight, turn around, or turn right or left (Brown & Heathcote, 2008). The information sought might involve specific environment landmarks (*landmark information*), how these landmarks relate to one another (*configural information*), whether the landmarks or structures together form an overall pattern (*geometric information*), or a combination of these information types*.* Together, landmark, configural, and geometric information form the foundation of our spatial memories (Montello, 1998; Siegel & White, 1975). Spatial cognition research reveals that multiple factors affect attention to and, consequently, processing of different types of environmental information (e.g., Brunyé & Taylor, 2009). These factors may help inform understanding of how spatial uncertainty affects information gathering.

### 3. Uncertainty

*Uncertainty* is a mental state experienced when attempting to decide between two or more competing choices. Decisions under uncertainty can involve either known or unknown probabilities. Known probabilities have been referred to as *risk* (Bernoulli, 2011) and unknown ones as *ambiguity* (Ellsberg, 1961). Uncertainty has been studied with regard to cognition broadly and navigation more specifically. As our opening example illustrates, uncertainty is prevalent when we navigate, particularly in new environments.

Uncertainty can arise during navigation for several reasons. First, it can arise when one attempts to orient oneself in a relatively unfamiliar environment. For instance, when exiting a subway station, gaining an initial orientation can be challenging and elicit uncertainty (Ishikawa & Yamazaki, 2009). Second, uncertainty can arise when one selects initial path segments and plans a route toward a destination. For example, once a navigator has successfully oriented, he must plan a route by selecting initial and subsequent path segments from among multiple possibilities; these options can elicit uncertainty (Wiener, Lafon, & Berthoz, 2008). Third, uncertainty can arise during wayfinding when a navigator seeks feedback about progress along a route. For instance, if a distal landmark becomes imperceptible, such as being blocked by a building as one walks, a navigator may become uncertain about whether she is still on course (Burgess, Doeller, & Bird, 2009).

When uncertain, we take steps to reduce uncertainty, one of which likely involves information gathering (Bruner, 1973; Kuhlthau, 1993). Uncertainty’s impact on information gathering has been considered within the contexts of knowledge construction (Bruner, 1973; Dewey, 1933; Kelly, 1963) and information search (Chowdhury, Gibb, & Landoni, 2011; Kuhlthau, 1993). When uncertain about which path leads to your desired destination, randomly selecting one route without additional input does not seem like a productive strategy. Gathering more information from the environment could differentially weight the choices, thereby decreasing uncertainty and increasing navigation efficiency. Actions such as looking around the environment, searching for distinguishable landmarks, or pulling out a map are behaviors that likely reflect information gathering. Further, these actions could be used as behavioral uncertainty metrics, i.e., marking points to examine what information individuals seek. Importantly, having finer-grained uncertainty metrics and/or metrics prior to outcomes could reveal what informs these navigation choices. In turn, responses to uncertainty, whether cognitive or affective, could help inform its role in navigation.

### 3.1. Uncertainty: measurement in context

Understanding how uncertainty affects cognitive behavior requires clear uncertainty indicators. Tolman (1948) used rodents’ pause-and-look behavior as a starting point for further exploring navigational processing. In a similar vein, if uncertainty can be clearly identified in humans, then actions taken in response can be evaluated. Aiming to determine potential behavioral measures of uncertainty, Brunyé, Haga, Houck, and Taylor (2017) focused on a common behavior that people exhibit when seemingly lost: looking around. An individual highly uncertain about his current location would likely look around more than a person who is certain. To capture this behavior, the researchers continuously recorded heading direction during virtual environment (VE) navigation. The continuous heading data were analyzed in two ways, using circular variance (variability around the person) and entropy (the probability of adopting various headings). While both techniques evaluate looking-around behavior, heading entropy showed a stronger relationship to path efficiency, a proxy of navigation success. Higher entropy (looking in more directions) coming into intersections, the point in an environment where a decision needs to be made, related to decreased path efficiency (Brunyé et al., 2017). This suggests that looking around could signal navigational uncertainty, particularly at decision points, and subsequent actions could reflect responses to uncertainty. Further, quantifying looking around with entropy appears to predict future navigation success.

*Entropy*, as a general term, is the amount of disorder or uncertainty about a state or situation (Shannon, 1948). In other words, the more variability in a behavior, the more uncertainty there may be. While this definition of entropy originated as an engineering construct, it has been used to indicate the degree of uncertainty in a range of behavioral, biological, and psychological contexts, including navigation (e.g., Brunyé et al., 2017; Hirsh, Mar, & Peterson, 2012). As one example, entropy has been used to measure inattention in driving. Using steering wheel variation, or steering entropy, higher entropy is related to more car crashes (Boer, 2000, 2001). The work reveals that behavioral entropy reflects behavioral variations well and in so doing is considered a viable quantitative metric for transient states of inattention. Within biology, entropy is discussed in terms of internal disorganization or uncertainty that must be reduced to ensure an organism’s survival (Prigogine & Stengers, 1997).

This description leads nicely into the psychological entropy definition provided by Hirsh et al. (2012), where entropy is anxiety-provoking uncertainty that must be managed to prevent potential negative consequences. Due to the potential stresses of psychological entropy, Hirsh et al. (2012) proposed a theoretical framework known as the entropy model of uncertainty (EMU). The EMU aims to understand the impact entropy/uncertainty has on not only an individual’s neural firing, but also on behavior. We will describe the EMU in more detail in the subsequent Uncertainty: affect and cognition subsection. Taken together, this work suggests that task-relevant behavioral variability relates to task uncertainty. With navigation, looking around can accurately indicate navigation uncertainty (Brunyé et al., 2017). We suggest using behavioral variability beyond simply measuring uncertainty. Specifically, we propose using increases in behavioral variability to mark important time frames to further explore how uncertainty affects actions and related cognitive behaviors. For navigation, this could include using increased looking around (denoting feeling lost) to then examine what information people further process. The information sought likely relates to how spatial mental models develop.

Changes in behavioral variability are likely not limited to a single behavior. To date, navigation uncertainty has been examined by considering looking around, operationalized as changes in heading direction. There are likely other measures of this transient uncertainty state, such as changes in walking speed or specific eye movements, as well as neural measures such as pupil diameter or physiological and neurophysiological responses (Brunyé & Gardony, 2017; Cavanagh et al., 2011; Thayer, Åhs, Fredrikson, Sollers III, & Wager, 2012; Urai, Braun, & Donner, 2017). In fact, the EMU proposes that entropy arises in neural systems when there is conflict between competing perceptual information and behavioral options (Hirsh et al., 2012). Entropy in overt behavior, such as erratic looking behavior, may reflect the inherent disorganization of mental processes during uncertainty and its accompanying anxiety. For example, recent research in the medical domain examines whether eye movement entropy may prove valuable for monitoring workload levels and uncertainty among surgical trainees (Di Stasi et al., 2016). Next we examine how uncertainty has been examined in other cognitive contexts and how a continuous behavioral measure would inform the outcomes of those contexts.

### 3.2. Uncertainty: affect and cognition

Uncertainty is often accompanied by an affective response. Continuously measuring affective responses (e.g., skin conductance, heart rate, etc.) could be one way to measure uncertainty throughout a scenario. As proposed by the EMU, affective responses to uncertainty are linked to four primary mechanisms and processes (Hirsh et al., 2012). First, uncertainty states pose an adaptive challenge that decision-makers are constantly seeking to manage and minimize. Second, conflicts between environmental cues and task-related behavior produce transient uncertainty states. Third, expertise in a domain helps decision-makers adopt clear goals and decision criteria that then help reduce uncertainty in the face of conflict. Finally, anxiety is the subjective manifestation of uncertainty states, associated with measurable neural (anterior cingulate) and hormonal (noradrenaline) responses. The EMU offers a framework through which we can understand uncertainty, examine the behaviors underlying uncertainty, and therefore behaviorally measure uncertainty.

Affective responses to stress differ in both valence and arousal depending on the nature and potential consequences of the uncertainty. High-stakes consequences, even in the absence of known probabilities, more often lead to negative affect (e.g., Slovic & Peters, 2006). In fact, when uncertainty heightens a risk appraisal, it can change intentions and behavior (Sheeran, Harris, & Epton, 2014), and capturing this behavior change should have utility for examining behaviors that follow it. Navigational uncertainty involves ambiguity, more so than risk, and a common response to ambiguity is aversion (Slovic & Tversky, 1974)*.* People who have high anxiety related to small- and large-scale spatial tasks (i.e., spatial anxiety; Lawton, 1994) feel as though many of their spatial decisions are ambiguous and show aversion to (and avoidance of) novel spatial experiences altogether (Gagnon & Wagner, 2016; Gunderson et al., 2013). People also differ in their tolerance for uncertainty; individuals having less tolerance show more negative affect, such as worrying (Dugas, Gosselin, & Ladouceur, 2001). Therefore, when faced with ambiguity during navigation, individuals may completely avoid the situation causing the uncertainty or worry as a result of that uncertainty. Avoiding the uncertainty may result in slowing the speed of approach or retracing their steps. Additionally, worry or anxiety that may result from the uncertainty could lead to measurable physiological responses (e.g., increased heart rate, change in skin conductance, etc.). Arousal related to an affective response has also been shown to impact how navigators process or learn an environment. During learning, when presented with a stressor (time pressure), individuals learned local landmarks (only one building present at a time) more accurately than global landmarks (multiple high-rise skyscrapers visible simultaneously; Credé, Thrash, Hölscher, & Fabrikant, 2019). This suggests that, when aroused, people rely more heavily on landmarks than landmark configurations. Other research has also examined aversion, which often accompanies arousal, but the context (how risky or ambiguous a situation is) matters (FeldmanHall, Glimcher, Baker, & Phelps, 2016; Kállai, Karádi, & Feldmann, 2009). Relating their results to navigation, this could mean that when an individual is highly uncertain about which direction to choose to get to the store, and is not highly aroused due to threats or time constraints, she would be more likely to make a risky choice (e.g., wander around until she finds it). This could increase behaviors (e.g., wandering around, looking around, seeking a map, etc.) leading up to arriving at the desired destination. Such behavior would not be accounted for in typical behavioral measures of navigation, which often only focus on navigation outcomes. However, if an individual were highly aroused, he might be less likely to engage in a risky behavior and instead seek a map or ask help from a passerby. As such, their overall behaviors would be different. Map seeking would increase, but other behaviors, such as distance traveled, would be reduced (i.e., the person could more directly navigate to the destination based on the direction information he/she acquired). Based on navigation time, it may seem that the person was less uncertain, but considering other continuous behavioral measures could reflect uncertainty and actions taken to reduce it, such as accessing a map.

Further, arousal, more so than valence, relates to memory impairment (Corson & Verrier, 2007) and a tendency to process categorically (Friedland, Keinan, & Tytiun, 1999). Important for navigational uncertainty, Brunyé, Mahoney, Augustyn, and Taylor (2009) found that arousal while learning an environment led to a greater configural focus (looking at how information relates and creating a larger picture) at the expense of landmark processing. Other research, however, shows that arousal narrows attentional focus (e.g., to landmarks; Reisberg & Heuer, 1992). Regardless, arousal related to uncertainty appears to shift cognitive processing by changing the information focus. Task demands likely impact how arousal shifts attention. Some evidence suggests that arousal during online processing (e.g., perceptual processing of landmarks) narrows focus to landmarks, but arousal during offline processing (e.g., memory retrieval) leads to a configural or geometric bias (Brunyé et al., 2009). These findings would predict that information seeking would focus on bigger picture (configural or geometric) aspects of an environment if relying on memory, but a smaller picture (landmark) focus while within the environment. Furthermore, the nature of the landmarks, whether proximal or distal, also impacts this focus. Distal landmarks inform an organism’s orientation within an environment, but when proximal and distal landmarks are in conflict, proximal landmarks are preferred (Knierim & Hamilton, 2011). Continuously measuring behavior or arousal during uncertainty could further pinpoint what is happening at each moment in time and subsequently what information is sought to reduce uncertainty.

Uncertainty likely impacts cognitive processing beyond that related to an affective response. However, research has not extensively addressed uncertainty’s role in cognitive processing (e.g., attention, working memory, cognitive load, information gathering, etc.). Some work suggests that uncertainty detrimentally affects cognitive processing, reducing attention and decreasing encoding. Uncertainty increases cognitive load, often engaging working memory resources (Coutinho et al., 2015) and activating a complex network of brain responses to up-regulate vigilance and information gathering (Brunyé & Gardony, 2017; Heekeren, Marrett, & Ungerleider, 2008; Payzan-LeNestour & Bossaerts, 2012). The cognitive load reduces our ability to use those cognitive resources for concurrent tasks while uncertain and can lead to resource depletion for subsequent tasks (Coutinho et al., 2015). In contrast, uncertainty may be beneficial for some cognitive processes. Uncertainty promotes goal-driven information seeking (Gottlieb, Oudeyer, Lopes, & Baranes, 2013). It also appears to promote metacognitive processing; when ambiguity is explicitly identified, both humans and monkeys can use this information adaptively (i.e., to avoid negative consequences; Shields, Smith, & Washburn, 1997). Finally, contextual variables likely play a role in how uncertainty impacts cognitive processing and therefore in how we navigate. As mentioned above, spatial navigation may be a good domain for exploring uncertainty’s cognitive impact given how frequently uncertainty arises when navigating a new environment (e.g., Brunyé et al., 2017; Stankiewicz, Legge, Mansfield, & Schlicht, 2006).

### 3.3. Uncertainty: navigational behavior

Uncertainty has been considered in how environments are learned and remembered. In robotics, one approach to modeling environmental learning involves accruing spatial knowledge into uncertainty or transformation matrices. Uncertainty/transformation matrices reflect estimated relations between location coordinates through a covariance matrix. Weights within the matrix reflect the uncertainty of these estimates (Smith & Cheeseman, 1986). As a robot navigates, these uncertainty matrices develop through experience in the environment; over time these matrices can be used to form Cartesian-like maps that can purposefully direct a robot’s movement (Cassandra, Kaelbling, & Kurien, 1996). Humans, like these robots, accrue information within an environment over time, combining the information into a spatial mental representation of their environment (Zeno, Patel, & Sobh, 2016). Uncertainty is inherent in much of this experience, emerging from navigation problems or errors (e.g., bumping into things, getting lost, etc.; Arleo & Gerstner, 2000). Uncertainty then prompts people to update their developing cognitive map (e.g., Arleo & Gerstner, 2000; Fleischer, Gally, Edelman, & Krichmar, 2007).

Similar factors related to an environment can impact both uncertainty and environmental learning success. Environment size impacts both (Stankiewicz et al., 2006). As environment size increases, uncertainty increases and navigation success (e.g., path efficiency) decreases. This relationship between environment size and uncertainty may arise for multiple reasons. Larger environments generally include more landmarks and concomitantly more landmark configurations, thus increasing the information needing to be encoded. With more information, it may be both more difficult to mentally update one’s mental map and more difficult to identify changes or missed or misremembered information. Continuous behavioral assessment of uncertainty could help pinpoint what information is being sought, if it is different between large and small environments, and what seems to contribute to navigational failure. Consistent with an inability to identify changes or misremembering information, uncertainty makes it difficult for people to update their mental map (Stankiewicz et al., 2006). Not updating, in turn, hinders future navigational success (Stankiewicz et al., 2006). Larger environments may also motivate people to process an environment strategically and configurally, similar to chunking (Gobet et al., 2001; McNamara, Hardy, & Hirtle, 1989). Instead of focusing on specific landmarks, people process the relationships between landmarks, including configurations, emerging geometry, and the interaction between geometry and landmark configurations. Evidence suggests that people group route segments into spatial chunks (Hirtle & Jonides, 1985; Stevens & Coupe, 1978). While grouping can facilitate learning, it sometimes interferes with memory for specifics (Roediger III & McDermott, 1995).

Multiple decision-making strategies combine for successful navigation (Schmidt, Papale, Redish, & Markus, 2013). Work with animals and humans reveals *place* and *response* strategies, each governed by a different brain area (Packard & McGaugh, 1996; Raiesdana, 2018). Place strategies involve flexible responses to environmental cues; response strategies combine rapid recognition with well-learned action sequences (Schmidt et al., 2013). Uncertainty may change navigational strategies and searching behavior. This has been observed in animal navigation, which is frequently presented as a good analog to human navigation. Typically, desert ants rely on visual memory (Wystrach, Mangan, & Webb, 2015) and path integration (Merkle, Knaden, & Wehner, 2006) to navigate back to their nest after foraging. However, visual memory and path integration are not without error, necessitating other approaches when they fail (Merkle & Wehner, 2010). When navigation errors arise, ants have high uncertainty about their nest location. At this point they adapt their behavior to use systematic search (Merkle et al., 2006) and/or rely on other cues, such as landmarks and scents (Wolf & Wehner, 2005). Other research has explored Bayesian integration in the context of ant navigation (i.e., Wystrach et al., 2015). Notably, the creation of an initial belief (prior) that is then changed (updated) based on evidence demonstrates that, when uncertain, ants weight path integration to a greater degree than other information, such as their visual memory (e.g., landmarks, landmark configuration, geometry of the environment, etc.) of their surroundings. The weight given to their visual memory is modified depending on their degree of uncertainty (Wystrach et al., 2015). This kind of result—that uncertainty triggers greater reliance on specific information, e.g., path integration for ants—suggests that examining information seeking under uncertainty can help us to better understand human navigation. Further, the knowledge that emerges can potentially be applied to developing navigational aids that help people get to their destination while still learning their environment and forming a mental map.

Similarly, rodents shift spatial strategies when uncertain (e.g., in cue conflict situations; Schmidt et al., 2013). Behaviorally, when these animals reach a decision point where cues conflict, they exhibit behaviors suggesting uncertainty, such as pausing and looking at navigation options, a behavior termed *vicarious trial and error* or *VTE* (Tolman, 1948). Recent work links VTE to hippocampal place cell firing, the pattern of which suggests consideration of navigation options (Redish, 2016). These findings parallel neural and behavior indicators for prediction or future option consideration (Buckner & Carroll, 2007; Schacter, Addis, & Buckner, 2007). When considering navigational options, an organism either needs to have a mental representation of the environment or needs to gather information to develop one. Further, as information is gathered and an organism learns about its environment, there is likely some Bayesian integration underway. When two forms of information occur simultaneously, they are probably weighted equally and therefore influence a decision equally. However, in situations involving prior experience and uncertainty, an organism relies more heavily on prior knowledge to determine what to do (Cheng, Shettleworth, Huttenlocher, & Rieser, 2007).

These results from desert ants and rodents suggest that animals change their strategies when faced with uncertainty. Desert ants, when uncertain, rely more heavily on path integration than on visual memory of their surroundings. Rodents display VTE, which suggests that they experience uncertainty and then either use their mental representation of their environment (visual memory like the desert ants) or seek information about their environment. While desert ants rely more on path integration when uncertain, rodents use Bayesian integration to determine what to do when faced with uncertainty. Bayesian integration relies more heavily on the rodents’ prior knowledge over new information. This work with animals offers insights into how human navigation may work and also motivates a need for continuous behavioral measures to examine human navigation. To understand what could go wrong when navigators face uncertainty, we must first understand how our spatial mental models (i.e., the map we have in our heads of how a city is laid out) develop and what is incorporated into them.

### 3.4. Uncertainty: summary

Uncertainty has been demonstrated to have both positive and negative outcomes on human behavior and affect, both within cognition generally and within navigational contexts. Within navigation, uncertainty may result in poorly formed spatial mental models, making subsequent navigation tasks difficult or potentially dangerous (e.g., ending up in a bad neighborhood). While individuals react differently to uncertainty, there may be common behaviors that can be measured to determine the degree of uncertainty and give an overall assessment of navigational performance. Continuous behavioral measures would reveal when uncertainty is present, how behavior changes due to uncertainty (e.g., different spatial information is sought), and how the outcomes change as a result. To begin to understand how uncertainty could impact the development of our spatial mental models, we first need to know how spatial mental models develop.

### 4. Spatial mental models

#### 4.1. Spatial mental model development

When uncertain, perhaps about one’s location in the environment or how to get to a destination, people likely access what they know about the environment from memory. By looking around, they could be trying to match what they see to what they remember and/or gathering additional information to further develop their spatial mental model. As such, theories and empirical data about spatial mental model formation would likely inform ideas about how uncertainty may influence information seeking. The types of spatial information people incorporate into memory include landmarks, landmark configurations, and geometric (routes, geometry, and other more complex configurations) information (Montello, 1998; Siegel & White, 1975). The timeline by which these information types are incorporated into memory remains a point of debate.

Two timelines describing spatial memory development based on navigation have been proposed. The sequential timeline suggests that this information is incorporated into memory serially (Siegel & White, 1975), starting by learning about individual landmarks and then connecting them into configurations that may eventually include the space’s geometry, first routes and then broader configurations. In other words, learning moves from landmark to geometric information. The simultaneous timeline suggests that landmark, configuration, and geometric information are learned in parallel (Ishikawa & Montello, 2006; Montello, 1998). However, cognitive and affective responses to uncertainty may affect the salience of and likelihood of incorporating these information types. These two timelines have implications for information seeking when uncertain. Siegel and White’s (1975) timeline suggests that people would seek landmark information, existing landmark knowledge, and turn-by-turn directions. Based on Montello’s (1998) timeline, people would seek all three information types.

We know, though, that people gather environmental information from multiple sources, in addition to what they can see while navigating. Maps and navigation aids are the most common navigation information sources. They involve a primary spatial perspective (egocentric or allocentric), which has implications for information seeking. An *egocentric* perspective, primarily available when navigating, involves locating environmental information relative to one’s own position. From an egocentric perspective, landmarks and local landmark configuration information is readily available. An *allocentric* perspective, primarily available with maps, involves locating landmark positions relative to each other from a viewpoint outside (generally above) the environment. From an allocentric perspective, all three information types (landmark, configuration, geometry) are readily apparent. Commercial navigational aids sometimes shift between these perspectives. However, without navigational aids, individuals may be forced to use one perspective (e.g., egocentric perspective because no map is available) over the other, even if they would be more confident or comfortable using information from the other perspective. As a result, landmark and landmark configuration information may be more salient over geometric information when uncertainty is present.

Thorndyke and Hays-Roth (1982) showed that spatial memory differed depending on how it was learned. Through navigation (egocentric perspective) people better remembered locations relative to their current position. From maps (allocentric perspective) people better remembered how landmarks related to other landmarks (landmark configuration and geometry). If the information source impacts what information people encode in memory, it likely plays a role in what information people seek when uncertain, dependent on whether one has a map available. Thus, if a person studied a map, but still remains uncertain about the path to take, she may seek information from another perspective (e.g., Dai, 2020). However, to know what information is guiding navigation when uncertain, we cannot rely solely on whether or not someone reaches the destination. Instead, we need to look continuously at what the person is doing while trying to get to the destination. This information can help determine what is guiding that person’s navigation.

#### 4.2. Using spatial mental models

How people learn about an environment affects their spatial memory, which in turn has implications for how they later think about that environment or what they can mentally do with that information (e.g., Brunyé, Rapp, & Taylor, 2008; Brunyé & Taylor, 2008; Taylor, Naylor, & Chechile, 1999; Taylor & Tversky, 1992). Thorndyke and Hays-Roth (1982) explored differences in environment knowledge based on two main information sources: navigation and maps. Maps, which present a survey perspective, provide landmark, landmark configuration, and geometric information directly. Through navigation, which provides a route perspective, landmark information is readily evident, but landmark configural and geometric information require time and experience in the environment. Brunyé et al. (2008) examined the role of these information types by giving participants either survey (e.g., *Johns Park is located on the northeast side of Whited Street.*) or route descriptions (e.g., *Turn right directly onto Whited Street. Johns Park is on the right side of Whited Street.*). Participants receiving survey information took less time to learn and had greater representational flexibility, defined by the ability to access landmark and geometric information to answer questions, than participants who received route information. Brunyé and Taylor (2008) suggest that survey knowledge is more readily abstracted into spatial mental models, while route knowledge requires more experience and information integration before the knowledge can be abstracted.

The ability to use a spatial mental model, the map of an environment one forms in one’s mind, adaptably (e.g., follow a route from a map or draw a map after navigating) indicates *representational flexibility*. Representational flexibility also predicts how effectively people use spatial mental models (Brunyé et al., 2008). The level of abstraction or representational flexibility of one’s spatial mental model likely interacts with uncertainty when within an environment. Someone with a less abstracted spatial mental model would likely still be building a geometric framework and would first seek landmark information. Someone with a more abstracted spatial mental model would likely have less uncertainty, but if faced with uncertainty may seek configural or geometric information. These ideas motivate methodologies and hypotheses for continuing research. In a similar way, Bilge and Taylor (2010) demonstrated that not only are map and navigation learning better suited to different sized environments, large and small respectively, but we also mentally update as we navigate. Instead of “here” (i.e., the place you start on a map: “You are here”) remaining stationary, it changes as you move. Further, “here” varies depending on the environment size and could encompass multiple entities (e.g., the chair in a room or the house you are in or the city in which the house is located; Bilge & Taylor, 2010). This result supports the need for a continuous measure of uncertainty, as the “here” identified at the beginning of navigation is unlikely to be the “here” at the end. Without a way to continuously identify “here”, information about what “here” is at each time point in navigation is hidden from the navigational process.

How people plan to use spatial information, or their goal, also affects their spatial memory. Taylor et al. (1999) had people learn an unfamiliar building either from a map or via navigation with either a route-based (learn the fastest route) or a configuration-based (learn how the rooms relate to one another) learning goal. Participants incorporated both source-based and goal-relevant information. For instance, if one wanted to look at a map to figure out how to get to a friend’s house (route goal), one would likely remember information about the route to the house plus geometric and configural information apparent on the map. In terms of information seeking when uncertain, people would more likely seek goal-consistent information (Brett & Vandewalle, 1999).

#### 4.3. Spatial mental models: summary

How we use spatial information to develop spatial mental models has been widely studied. Spatial information, as discussed here, can be subdivided into three categories: landmark, landmark configuration, and geometric. Yet, when navigational uncertainty occurs, the spatial information sought varies based on the context of that situation, which then alters spatial mental model development. As individuals try to reduce uncertainty, they may seek spatial information they deemed reliable in past spatial mental model development, or they may look for a different spatial information type. This process of seeking out specific information to reduce uncertainty inherently changes the spatial mental model being developed and the navigational process used to reach the desired destination. By using continuous behavioral measures, we can examine what is happening moment to moment, how each information type is being weighed, and how that information is incorporated into the final spatial mental model.

### 5. Continuous behavioral and neural measures

Navigation, here defined as the process of taking a known path or finding a way through an unknown environment (i.e., wayfinding), is a continuous process, meaning we do not instantly know our way around a new environment or necessarily know the best way to get between points A and B even in a familiar environment. We are constantly learning new things about our environment as we interact with it, but when we measure navigation success, we generally examine discrete measures (e.g., time to completion, path efficiency, or success in reaching a target location). These discrete measures gloss over many behaviors in which people engage while navigating. Some spatial cognition research has begun to use continuous measures, some of which have previously been applied to other cognitive processes. This work suggests that continuous measures can provide more insight into cognition generally and thinking about spatial environments specifically. Both behavioral and neural continuous measures have been used.

Commonly used continuous behavioral measures for exploring cognitive processes include mouse tracking and eye tracking. Tracking computer mouse position while someone makes a choice (e.g., one route versus another) has been used to infer cognitive issues used to make that choice (Freeman & Ambady, 2010). Wang, Taylor, and Brunyé (2019, 2020) used mouse tracking to show how actual spatial location interacted with the linguistic term used to describe spatial location when verifying a spatial description (e.g., “theater to the west of the restaurant”). Mouse tracking used with this action-compatibility paradigm revealed that the linguistic term had a stronger impact on the verification decision when the environment was not learned as well. This finding would not have been apparent without the continuous data. Eye tracking (Hollander et al., 2019) also provides continuous data. Eye-tracking metrics have been used to reflect attention to particular information. These include dwell time and shifts between regions of interest, among others. Kiefer, Giannopoulos, and Raubal (2014) demonstrated that successful navigators focus more visual attention on map symbols relevant to their destination compared to unsuccessful navigators (who may look at multiple symbols). Using eye tracking and/or virtual reality (VR) within a spatial reorientation context would allow researchers to see if the same holds true when faced with uncertainty, whether fixation or dwell time are longer for certain environmental components (e.g., landmarks, geometry, etc.), suggesting those components inform individuals’ decision processes when facing uncertainty. Any of these measures have advantages and difficulties and should be carefully selected based on their feasibility and relation to the cognitive construct and task at hand. In addition to those described above, navigation-specific behaviors (e.g., heading change, environment location) can be collected in a continuous manner (Brunyé et al., 2017). Collecting these measures is already straightforward in virtual environments.

Neural continuous measures, including electroencephalography (EEG) and functional near-infrared spectroscopy (fNIRS), have been used to explore a variety of cognitive processes. Here we focus on a few studies where these neural measures have been examined in spatial contexts. EEG has been used to examine neurally based electrical changes at the scalp before, during, and after a VR immersion (Kim, Kim, Kim, Ko, & Kim, 2005). This work showed a relationship between VR immersion and EEG delta and beta wave activity. In the past, EEG recordings have been too noise prone to allow locomotion during recordings. However, recent research by Gramann and colleagues has demonstrated that not only does mobile brain/body imaging (MoBI) reveal no differences between standing, slow walking, and fast walking conditions (Gramann, Gwin, Bigdely-Shamlo, Ferris, & Makeig, 2010), but using a regression procedure and spatial filtering allows noise from human gait to be removed from EEG data (Gwin et al., 2011). These results suggest that EEG could be used, not only in a VR desktop setting, but also with immersive VR. This would allow any cognitive differences arising from physical movement, as opposed to movement through a mouse and keyboard, to be revealed. EEG data would enable a direct comparison between the immersive and desktop VR navigation. fNIRS measures brain activity in the prefrontal cortex (PFC; Holtzer et al., 2011). Given where it measures, it generally reflects executive function-related processing, particularly cognitive load. fNIRS has been shown to reflect changes in a person’s gait during navigation, and gait changes while talking have further been linked to uncertainty (Spiers & Maguire, 2006, 2007). Functional magnetic resonance imaging (fMRI) has begun to examine dynamic continuous behavior in real-world settings by teasing out neural activity related to specific events (Spiers & Maguire, 2007). These data reveal the roles brain areas play during navigation (Spiers & Maguire, 2006); however, fMRI has mediocre temporal resolution, so exact structures are hard to pinpoint. While these continuous neural measures reveal what the brain may be doing over time, discussing them in the context of available continuous measurements is important.

In this paper, we primarily focus on how continuous behavioral measures can provide insights into information processing during navigation. As a starting point for examining what continuous navigation measures can tell us about information gathering during navigation uncertainty, we describe in detail one oft-used spatial navigation paradigm: spatial reorientation (e.g., Cheng, 1986; Sturz & Kelly, 2009, 2013).

### 5.1. Spatial reorientation paradigm

*Spatial reorientation* may be a good starting paradigm to systematically look at the information continuous measures can reveal. Spatial reorientation is used to study the role of uncertainty and spatial navigation by focusing on the information one gathers during navigation and intentionally induces uncertainty while participants are learning an environment. The aim is to see what information types, landmark or geometric, people use to reorient and reach a goal. While the task itself does not continuously measure behavior, this task in conjunction with continuous behavioral measures offers a good, easily controlled environment from which to verify continuous measures of uncertainty and explore the utility of continuous navigation measures. While we see using this controlled paradigm as a good starting point, its elements can be seen in real-world navigation, as we will discuss throughout this section. Here we first describe the paradigm and then discuss how continuous navigation data within the paradigm could be insightful.

**Fig. 2:**
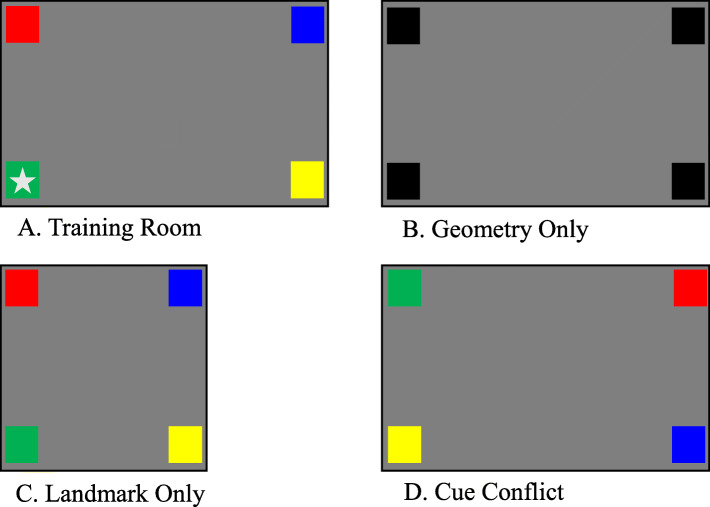
Spatial reorientation room layouts. **a** Depicts the layout of the training room with the *star* indicating the goal location. **b** Depicts the Geometry Only test condition where there is no landmark information available for participants to utilize. **c** Depicts the Landmark (formerly Feature) Only test condition, where geometric information has been removed. **d** Depicts the Cue Conflict test condition, where the landmark information is rotated by one corner so that the landmark and geometric information are no longer corresponding to the training room

The basic task places participants in a rectangular room that contains four landmarks (e.g., four colored boxes). A landmark is placed in each corner, and one corner is designated the goal location. The participants are told to locate the goal (i.e., find an object hidden in one corner of the room), but are not given instructions about what information to use. The goal (indicated in Fig. 2a by a gray star) is not readily visible; this ensures that participants will explore and learn about the room. Thus, the room includes landmark and geometric cues. The room’s shape (rectangular) serves as a geometric cue for the goal (e.g., the goal is where the short wall on the right meets the long wall on the left). The corner objects serve as landmark cues. During the task, participants are disoriented in each trial. With humans in a real-world environment (rectangular room), disorientation involves blindfolding the participants while spinning them to face a random direction. Disorienting participants in this way increases uncertainty (for a review, see Cheng, Huttenlocher, & Newcombe, 2013). The paradigm includes learning and test phases. When learning, participants typically have unlimited time and opportunities, within a trial, to explore the room and find the goal (e.g., Lee & Spelke, 2008; Lourenco, Addy, Huttenlocher, & Fabian, 2011; Reichert & Kelly, 2011; Sturz & Kelly, 2009, 2013). Learning continues for a fixed number of trials or until the participant can reliably find the goal location after disorientation (cf. Cheng & Newcombe, 2005). During learning the goal location is visible, so participants merely need to find it. However, during testing there is no visible goal location, so participants must make a choice about which corner they believe is the goal location, based on their experience. Like most navigation studies, spatial reorientation generally involves a discrete measure of performance. We propose using continuous behavior measurements with this paradigm to learn more about the information used to make their final choice.

Analogs to the spatial reorientation paradigm can be found in the real world. For example, imagine starting a new job and taking the subway home from work every day. There is a learning phase during which you learn that coming out of the Main St. exit results in your finding a Dunkin’ Donuts on the left and a sushi restaurant on your right. From repeated visits you know to go left toward the Dunkin’ Donuts and your apartment is just a block away. However, your knowledge of the environment is tested when the Main St. exit is closed for construction and you exit through Center St. Here, you discover another Dunkin’ Donuts and an office building. What do you do in this situation? Here the landmark information is misleading (i.e., another Dunkin’ Donuts), but examining the landmark configuration should reveal that it is not the same path you normally take. Alternatively, you could rely on the geometry of the subway station and its relative position compared to surrounding roads. How do you decide where to go? What information do you use to navigate home? The spatial reorientation paradigm addresses these exact questions and offers a controlled means for testing what our continuous behavior reveals about the information we seek and the navigation behaviors we engage in when faced with uncertainty.

The three test conditions are designed to assess which information individuals use to code the goal location. The *Landmark Only* (also known as Feature Only) test changes the room geometry from a rectangle to a square, removing geometry as a cue (see Fig. 2c). If participants select the landmark that was associated with the goal during training, then they have encoded landmark information. If they have only encoded the room’s geometry, then they will randomly select a goal. Research shows that adults (e.g., Kelly & Bischof, 2008; Reichert & Kelly, 2011; Sturz & Kelly, 2009, 2013) and children (e.g., Lee, Sovrano, & Spelke, 2012; Lee & Spelke, 2008; Lourenco et al., 2011) can readily use landmark information when geometry is unavailable. The *Geometry Only* condition uses the rectangular room, but makes landmark information uninformative. For example, landmarks may be removed altogether or made perceptually identical (e.g., colored boxes are now all black; see Fig. 2b). In the Geometry Only condition, if a participant has encoded geometry, he would either choose the trained corner or the corner diagonal from it. These two corners are geometrically equivalent. With uninformative landmarks, selecting either corner suggests use of the available geometric information. Results suggest that both adults (e.g., Kelly & Bischof, 2008; Reichert & Kelly, 2011; Sturz & Kelly, 2009, 2013) and children can use geometric information, implying it is salient across the lifespan (e.g., Lee et al., 2012; Lee & Spelke, 2008; Lourenco et al., 2011).

The most intriguing test is the *Cue Conflict* condition. In the Cue Conflict condition, uncertainty is created by placing geometric and landmark information at odds (see Fig. 2d). To do this, landmark information is shifted, rotating it (clockwise or counterclockwise, direction is irrelevant) one corner relative to the original rectangular training room. By doing so, the landmark no longer corresponds with the geometry experienced during training. This cue conflict creates high uncertainty; previously both landmark and geometric information identified the goal location, and now the participant must choose between these information types. This condition can reveal which information is more likely used. If participants use geometric information, they would choose one of the geometrically equivalent corners; if they use the landmark information, they would select the trained landmark. Bayesian theory suggests that people use past experience when facing uncertainty (Weise & Woger, 1993). In the Cue Conflict condition, however, a participant’s past experiences included both landmark and geometric information, so neither information type can be reliable (Cheng et al., 2007). Responses then suggest whether someone relies on landmark or geometric information to a greater extent.

The spatial reorientation paradigm has been used with both humans (across age categories) and other animal species. Combining these two literatures guides understanding of which cues people either integrate into their mental model or favor during uncertainty. Focusing on the Cue Conflict condition, the spatial reorientation results show that different species and the same species at different ages use different cues (see Table 1). Rats (Cheng, 1986) and fish (Sovrano et al., 2003) prefer geometric over landmark information. Specifically, these animals incorrectly search a geometrically symmetric location, even in the presence of predictive landmark cues (e.g., Cheng, 1986). Based on such findings, the presence of a *geometric module* for orientation has been proposed and debated. The debate arises because other animals, like pigeons (Kelly et al., 1998), chicks (Vallortigara et al., 2004), and rhesus monkeys (Gouteux et al., 2001) prefer landmark information, even without training. Human adults also preferentially rely on landmark information (e.g., Kelly & Bischof, 2008; Reichert & Kelly, 2011; Sturz & Kelly, 2009, 2013). Human children, like rats and fish and unlike human adults, prefer geometric information (e.g., Lee et al., 2012; Lee & Spelke, 2008; Lourenco et al., 2011). They can use landmark information if it is the only information available (e.g., Landmark Only condition; Hermer & Spelke, 1996). With this variability, it is difficult to conclude that one information type supersedes the other when a person is either uncertain or needs to reorient. Thus, knowing more about spatial information seeking is important to understand how we navigate and update our mental models of an environment.

**Table 1:** Spatial reorientation Cue Conflict results across multiple species

Citation	Population	Landmark (route)	Geometric (survey)
Hermer and Spelke (1996); Learmonth, Newcombe, Sheridan, and Jones (2008); Lee and Spelke (2008)	Human children aged 18 to 24 months Human children aged 3, 4, 5, or 6 years Human children aged 4 years	No Only in large environments No	Yes Yes Yes
Kelly and Bischof (2008)	Human adults	Yes	No
Gouteux, Thinus-Blanc, and Vauclair (2001)	Rhesus monkeys	Yes	No
Cheng (1986)	Rats	No	Yes
Sovrano, Bisazza, and Vallortigara (2003)	Fish	No	Yes
Kelly et al. (1998)	Pigeons	Yes	No
Vallortigara, Pagni, and Sovrano (2004)	Chicks	??	??

^??^indicates that the results were inconclusive/mixed

The primary behavioral response with the reorientation task is location selection (i.e., a single, discrete choice). While it shows which information type “won” in a participant’s selection, it provides only one data point per trial. Continuous behavioral measures could reveal the extent to which a participant considered other information. Animal research has begun to extend beyond choice data to examine neural responses when cues conflict. Julian, Keinath, Marchette, and Epstein (2018) reviewed research surrounding the cognitive and neural underpinnings of the spatial reorientation paradigm. Their review links rodent spatially tuned cells to human neurobiology, suggesting that continuous neural measures might provide insights into navigation under uncertainty. Important for the current work, neural responses are collected on a continuous basis while the animal completes a task, in this case locating spatially organized goal locations (Julian et al., 2018). While a continuous measure of human spatially tuned cells has not been collected (and is not possible with current methodologies) during a spatial reorientation task, the data from rodents suggest that we can gain greater insights on cognitive processes underlying navigation from continuous measures. We suggest that collecting continuous behavioral data could provide insights into what information people consider when uncertain.

As an example, implementing this paradigm in a virtual environment, the continuous measures (through mouse tracking or physical location) that could be examined are heading direction (how much are you looking around versus maintaining a consistent heading?), speed of travel (are you slowing, speeding up, or maintaining your pace?), and approach angular difference (what is the difference between the ideal angle at which you should approach your target destination versus how you actually approached?). Speed of walking or travel can be likened to Tolman’s (1948) research on pausing and looking behavior during VTE. In a VR environment this speed of travel could indicate when a participant slows down, stops, or pauses, which would be indicative of uncertainty as well as any navigation options being considered. While she is stopped, we could examine the heading to see if she is looking around for additional cues. If the headings are clustered toward geometric or landmark information, the extent to which she looked while walking toward the other options suggests that she is still processing and/or reassessing the options. Finally, her approach angular difference, a moment-by-moment comparison between the ideal angle of approach (given their current location) to the actual angle of approach, could reveal if she is exploring other areas of the room first before approaching the goal location. These measures could translate just as easily to the real-world example mentioned above, with speed of walking, angular difference, and heading direction indicating how confident you are when navigating home, whether you considered the changed landmarks (i.e., no sushi restaurant), or whether you just raced home, ready to relax after a long day.

A challenge of using continuous measures is in analyzing such both rich and dense datasets. We can draw on some existing analysis approaches. Mouse-tracking work provides some data analysis (Freeman & Ambady, 2010). As in VR, mouse tracking collects continuous *x-* and *y*-coordinates of the computer mouse location while participants move it relative to multiple response options. From these data, a number of informative measures can be calculated, including spatial attraction/curvature, motion complexity, velocity, and acceleration. Similarly, by collecting the *x-* and *y*-coordinates of avatar position in the VR environment, measures of proximity to target location options, speed of locomotion (including pauses), and changes in direction over time can be assessed. Analogs of eye-tracking metrics can also be used, in this case examining heading data. With eye tracking, metrics of fixation time, fixation shifts, and fixation sequences provide insights into information people are attending to and processing. Similarly, consistent versus changing heading directions over time can provide insights into both entering a state of uncertainty and the information types people consider when navigating.

There are several reasons to use the spatial reorientation paradigm as a starting point to explore the utility of continuous data. First, there is a wealth of data, with different age groups and species using this paradigm. This allows for comparison and extension of well-replicated findings. Second, the paradigm purposely induces uncertainty by giving conflicting cue information during some test conditions. The Cue Conflict conditions would allow us to verify that heading change serves as an uncertainty signal (Brunyé et al., 2017). Because uncertainty is purposefully induced, examining other behavioral measures can potentially give insights into information people consider during the task. Third, the spatial reorientation has several methodological options for implementation, giving researchers options. These include using a physical room or virtual (VR) or augmented reality (AR). Each option has pros and cons. The same behavioral measures can be collected through each of the methods, but in different ways. The physical room allows people to navigate in the most natural way, by walking around. Movement, position, and heading data can be collected using cameras. However, coding camera data is time and labor intensive. VR and AR generally collect the relevant movement, position, and heading data automatically, lessening the coding burden. However, people generally have to be trained on how to interact with the environment, making it much less natural. Eye-tracking technology can be used in all of the methodologies, but some require wearable trackers.

### 5.2. Continuous behavioral and neural measures: summary

Examining navigation solely based on whether someone reaches their final destination (a discrete measure) oversimplifies the navigation process. When an individual is uncertain, he exhibits behavior moment to moment which reduces uncertainty and ideally leads to his target destination. Continuous behavioral (e.g., mouse tracking, eye tracking, etc.) and neural (e.g., EEG, fNIRS, etc.) measures could assess the level of uncertainty at any given moment as well as what information someone seeks to reduce that uncertainty. The spatial reorientation paradigm is a simple, well-controlled environment through which uncertainty is intentionally induced and continuous measures can be recorded within a VR environment. The simplicity of the environment will allow researchers the ability to develop measures of uncertainty and determine what information is being sought during uncertainty. This can translate into research in more complex and real-world environments, which could lead to fundamental changes in how navigational aids are developed.

6. Future directions

Navigational uncertainty, a cognitive state, leads to information seeking, which often involves observable actions. The information-gathering actions, in turn, lead to cognitive updating of one’s spatial mental model, which then guides navigational actions. This continuous interplay of cognition and uncertainty allows for environmental learning and successful traversal within the environment. While we know that uncertainty drives information seeking, the information sought remains unclear. Knowing this has important implications for navigational aid design, navigational instructions, and training methods to familiarize people with a new environment. In high-stakes situations, such as those involving emergency first responders, providing the right information as uncertainty arises could improve outcomes through reducing uncertainty-induced anxiety and strengthening associations between perceptual cues and behavioral affordances (Hirsh et al., 2012).

Continuous behavioral measures should provide a more fine-grained evaluation of how this cognitive state drives action. As stated previously, the spatial reorientation task provides an interesting means for studying uncertainty’s impact as it purposefully induces uncertainty. Further, this task has a wealth of discrete data from widely varied populations to which continuous data can be contextualized. To date, no one has used continuous measures, such as heading changes, path changes, or proximity to targets, with the spatial reorientation paradigm. Our lab has recently begun such experiments using desktop VR to evaluate the utility of continuous measures. We suggest that having behavioral indicators of uncertainty could mark critical junctures for examining which aspects of an environment people attend to as they develop a spatial representation. Heading entropy may provide such an indicator. Further, the continuous behavior itself should be informative. Similar to event-related paradigms (e.g., event-related potentials [ERPs] or event-related fMRI paradigms), presentations of a particular stimulus trigger the examination of and comparison between neural and behavioral responses. Within the spatial reorientation task, continuous heading, continuous location, and/or eye movement data can help identify whether an individual is attending to landmark, landmark configuration, or geometry during times of high uncertainty (Cue Conflict condition). It can then be compared to more certain trials (test in training room).

Continuous heading or path data can be used to reveal what information people are taking in. The primary behavioral measures would relate to movement within the environment. Taking heading measures, dwelling on a particular heading, and/or changing between certain ones should suggest the information under consideration. For example, heading dwell time and heading changes can reveal the extent to which a participant focuses on a specific corner with a specific landmark or considers two different corners, such as the one associated with the trained landmark and the one associated with the trained geometry. Likewise, these same measures can reveal whether someone strongly considers the room geometry, by alternately dwelling on particular long and short walls related to the trained geometry. The same logic can be applied to information related to landmark configurations. In a similar vein, location dwell time and movement changes can indicate information someone is considering. Akin to mouse tracking, movement toward one of the available cues, even if the location signaled by that cue is not eventually selected, would suggest consideration of that cue. Navigational speed could signal approach and/or avoidance of particular cues. Dwell time at a location more proximal to a landmark or geometric information could also suggest consideration of that information. Using continuous heading and movement data together will likely best help to interpret what the measures indicate. For example, in the Cue Conflict condition someone may walk toward the corner associated with room geometry during training. Here heading and movement data indicate consideration of geometry. The person may then stop partway and alternately look at this corner and the one now associated with the trained landmark, thereby taking landmark information into account. Movement may then shift toward the landmark-indicated corner, but the person may again stop and look toward the geometry-indicated corner. The continuous nature of these behavioral measures can inform understanding of dynamic mental processes that predict, indicate, and potentially resolve uncertainty during spatial tasks.

We consider the spatial reorientation paradigm as only a starting point for examining how uncertainty induces information-seeking actions. The paradigm is obviously simplistic relative to navigating in real-world environments. Once continuous measures can be validated in this and other more simplistic spatial tasks (e.g., the Morris water maze or radial arm maze; Astur, Tropp, Sava, Constable, & Markus, 2004; Vorhees & Williams, 2006), they could be explored in realistic virtual environments where the experimenter has some control over how uncertainty is induced.

If our current work validates heading entropy as a predictor of uncertainty and a signal for information seeking, the measure could be expanded beyond this simple spatial task to use in larger environments. The same is true for continuous changes in heading, movement, and location. Some of our suggested continuous measures would transfer readily to real-world environments. For example, return to our opening example of finding a grocery store in your new city. Upon leaving the bank, you can turn left or right. In deciding, you may turn and look for a bit in one direction then look in the other direction and then turn back to the first direction. Say you then decide to turn right, but then turn back and look in the other direction again, walk that way for a while, and then change your mind again. In the end, you find the grocery store along the road that was a right turn from the bank. However, all of your actions before finding the grocery store reflect information you considered along the way.

These results could be taken into account when relative to the negative impacts of navigational aids, which reduce uncertainty but also impair spatial memory development (Gardony, Brunyé, Mahoney, & Taylor, 2013). If the next generation of navigational aids takes into account what information people need and provides it when they exhibit uncertainty, could people both have the security that a navigational aid provides and also effectively learn the environment in which they are navigating?

## 7. Conclusions

Spatial navigation and learning are determined by what information is both available and encoded from the environment. Uncertainty dictates what information is examined and incorporated into our environmental mental models. We propose measuring continuous behavior, a pioneering method, to reveal how uncertainty impacts information sought during navigation. Current technology presents minimal uncertainty such that humans rely on it to an extreme, which hinders overall environmental learning. Measuring uncertainty using continuous behavior could be the change needed to shift technology back to a more assistive role: to supplement human learning and not replace it.

## Data Availability

Not applicable.

## Abbreviations

EEG: Electroencephalography
EMG: Electromyography
ERP: Event-related potential
fMRI: Functional magnetic resonance imaging
fNIRS: Functional near-infrared spectroscopy
IR: Infrared
LEC: Lateral entorhinal cell
MEC: Medial entorhinal cell
PET: Positron emission tomography
PFC: Prefrontal cortex
VE: Virtual environment
VTE: Vicarious trial and error
